# Real-Time Image Processing Toolbox for All-Optical Closed-Loop Control of Neuronal Activities

**DOI:** 10.3389/fncel.2022.917713

**Published:** 2022-07-05

**Authors:** Weihao Sheng, Xueyang Zhao, Xinrui Huang, Yang Yang

**Affiliations:** School of Life Sciences and Technology, ShanghaiTech University, Shanghai, China

**Keywords:** calcium imaging, closed-loop optogenetic stimulation, image registration, neuron segmentation, fast image processing, image alignment

## Abstract

The development of *in vivo* imaging and optogenetic tools makes it possible to control neural circuit activities in an all-optical, closed-loop manner, but such applications are limited by the lack of software for online analysis of neuronal imaging data. We developed an analysis software ORCA (Online Real-time activity and offline Cross-session Analysis), which performs image registration, neuron segmentation, and activity extraction at over 100 frames per second, fast enough to support real-time detection and readout of neural activity. Our active neuron detection algorithm is purely statistical, achieving a much higher speed than previous methods. We demonstrated closed-loop control of neurons that were identified on the fly, without prior recording or image processing. ORCA also includes a cross-session alignment module that efficiently tracks neurons across multiple sessions. In summary, ORCA is a powerful toolbox for fast imaging data analysis and provides a solution for all-optical closed-loop control of neuronal activity.

## Introduction

Establishing a causal relationship between neural activity and behavior is central to understanding brain function, and this endeavor has been greatly facilitated by optogenetics (Boyden et al., 2005; Deisseroth et al., 2006; Lutz et al., 2008; Zhang et al., 2010; Packer et al., 2012; Yang and Yuste, 2018). Currently, most optogenetic experiments use pre-determined stimulation protocols, without considering the ongoing activity of relevant neurons. However, given that neuronal activities are highly heterogeneous—closely linked to brain states and behaviors—employing real-time activity-dependent stimulation protocols will better reveal the dynamic interactions between neural circuits and behavior (O’Connor et al., 2013; Grosenick et al., 2015; Peters et al., 2017; Prsa et al., 2017; Zhang et al., 2018; Robinson et al., 2020). Such “closed-loop experiments,” in which the input to the system (i.e., optogenetic stimulation) depends on the output (i.e., neural activity), are now within reach owing to optical imaging techniques for monitoring ongoing neural activities (Tian et al., 2009; Chen et al., 2013). However, accurately identifying active neurons in real-time is an open challenge.

In principle, identifying active neurons from raw imaging data requires two essential steps. The first is image registration, to compensate for the shift between image frames, for which several algorithms are available (Thevenaz et al., 1998; Guizar-Sicairos et al., 2008; Dubbs et al., 2016); in this paper, we further accelerated this process by optimization. The second step and the major hurdle is to identify active neurons from the registered movies of ongoing activity. The current methods that feature either dimensionality reduction or deep learning are such that they require pre-processing of imaging datasets spanning a relatively long time for effective neuronal identification (Pnevmatikakis et al., 2016; Pachitariu et al., 2017; Mitani and Komiyama, 2018; Giovannucci et al., 2019; Soltanian-Zadeh et al., 2019; Speen et al., 2019), and are therefore unsuitable for this purpose.

In addition, these imaging analysis pipelines cannot automatically track the same regions of interest (ROIs) across multiple sessions. Imaging the same population of neurons *in vivo* for an extended period of time is now possible with genetically encoded indicators and the implantation of chronic imaging windows or GRIN lenses (Holtmaat et al., 2009; Driscoll et al., 2017; Peters et al., 2017; Xin et al., 2019), but manually identifying the same neurons from multiple imaging sessions is time-consuming and error-prone. Additionally, some microscopes allow the user to rotate the optical axis. This feature adds flexibility to *in vivo* imaging, but at the same time exacerbates the difficulty of identifying the same neurons captured with slightly different angles, as rigid transformation alone cannot correct such distortions (Sheintuch et al., 2017; Giovannucci et al., 2019).

**FIGURE 1:**
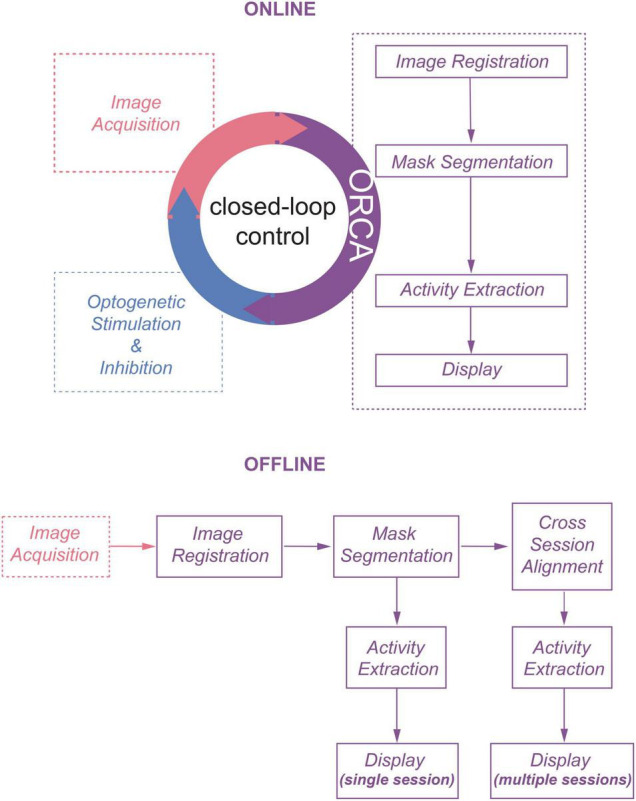
The online and offline pipelines of ORCA.

We envisioned that using statistical methods based on computing the temporal variance or deviation of the fluorescent signals of each pixel would allow us to achieve online identification of active neurons from streaming images. Pursuing this, we developed an image processing toolbox ORCA (Online Real-time activity and offline Cross-session Analysis), for fast image registration and online active neuron identification. ORCA also contains a cross-session alignment module for automatic tracking of neurons in long-term imaging (Figure 1). We demonstrate that ORCA can perform online identification of active neurons for closed-loop control, by identifying sound-responsive neurons in the mouse auditory cortex and selectively suppressing them with two-photon targeted optogenetic inhibition.

## Results

### Fast and Accurate Graphics Processing Unit-Based Image Registration

**FIGURE 2:**
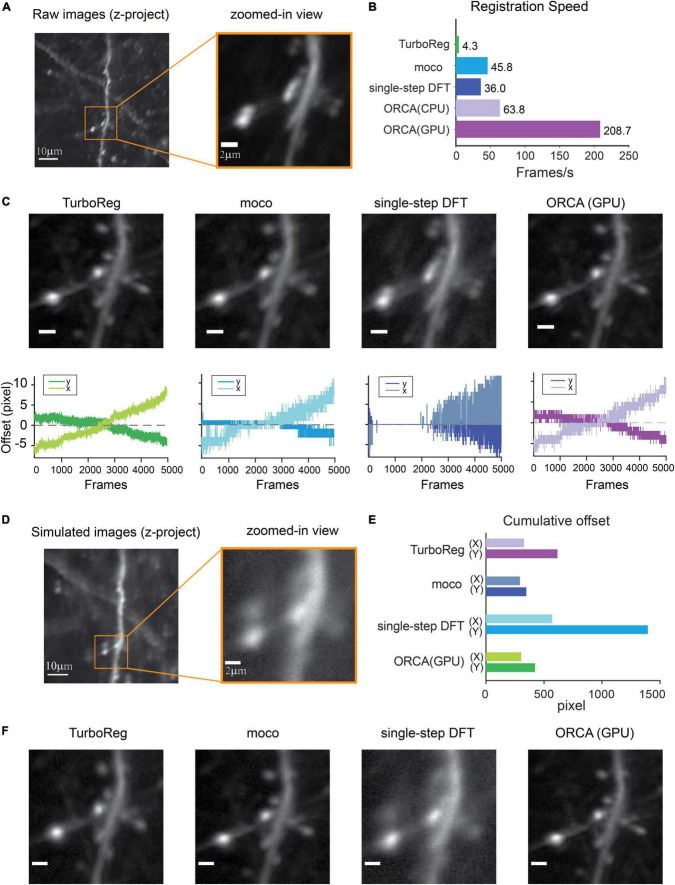
Raw image registration by ORCA and other software. **(A)** Z-project of raw images and zoom-in view. **(B)** Registration speed of different methods measured in frames per second. **(C)** Top panel: z-project of zoomed-in view after registration; bottom: calculated shifts in x and y directions. Scale bar, 2 μm. **(D)** Z-project of the simulated movie. **(E)** Quantification of registration accuracy with simulated data in panel **(D)**. **(F)** Z-project of zoomed-in view after registration of simulated movie. Scale bar, 2 μm.

The first module, *Image Registration*, removes motion artifacts caused by brain pulsation and body movement. To achieve high processing speed, we implemented an algorithm to search for the optimal translation optimized for Graphics Processing Unit (GPU) acceleration (see section “Materials and Methods” for details). We compared the processing speed of ORCA with single-step DFT (Guizar-Sicairos et al., 2008), TurboReg (Thevenaz et al., 1998), and moco (Dubbs et al., 2016), using an example movie of 5,040 frames of 512×512 pixels, containing both high-frequency lateral shifts and gradual drifting in the X–Y plane (Figure 2A). ORCA outperforms the other methods, especially when running on GPU (Figure 2B), with comparable registration accuracy as shown by the magnified z-stack images (Figure 2C, top panels). Examination of the offset of each frame relative to the template image indicated that ORCA captures both small and fast shifts, and large and slow drifts (Figure 2C, bottom panels).

To quantitatively assess the registration accuracy of ORCA, a simulated movie was created by compiling a series of identical images with pseudo-random x and y shifts, termed “true shift values” (Figure 2D). Image registration on this movie was conducted using ORCA and the other three methods mentioned earlier. ORCA achieves similar or better performance, as quantified by the discrepancy between calculated shifts and the ground truth, suggesting that ORCA is a more efficient tool in image registration (Figures 2E,F).

### Online Identification and Segmentation of Active Neurons

**FIGURE 3:**
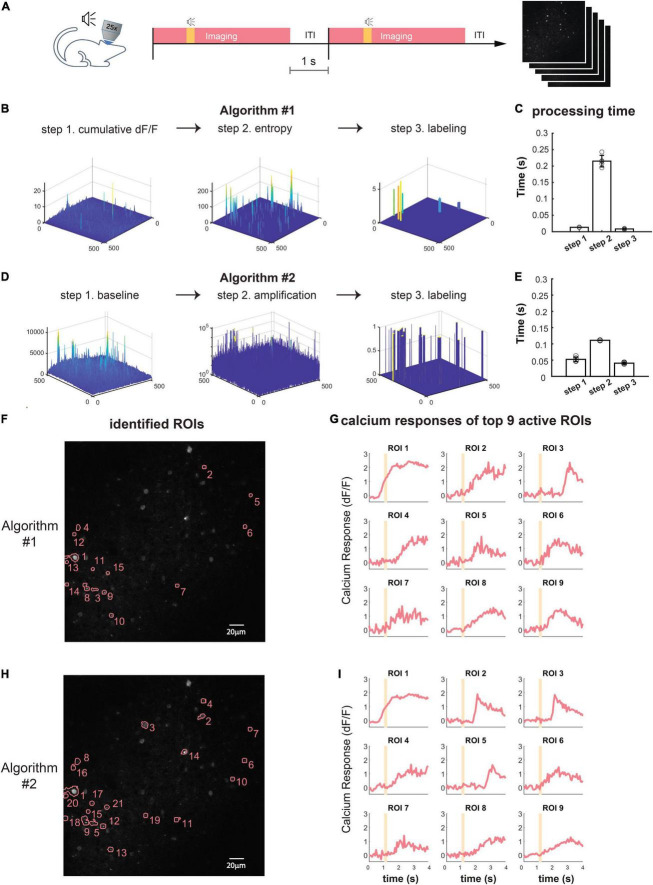
Online active neuron identification and activity extraction. **(A)** The flow of a typical trial-based imaging experiment. After 1-s baseline imaging, a pure tone lasting 0.2 s was played. Calcium activities were recorded for 4 s, and a 1-s inter-trial interval preceded the next trial. **(B,D)** Online neuron identification for each trial consists of three main steps for algorithm #1 **(B)** and algorithm #2 **(D)**. **(C,E)** Time spent in each step for processing data from one trial (60 frames) using algorithm #1 **(C)** and algorithm #2 **(E)**. Each data point represents the processing time for one trial. The average time is calculated for five trials. **(F,H)** An example of all identified ROIs by algorithm #1 **(F)** and algorithm #2 **(H)** in one trial. **(G,I)** Calcium responses (dF/F) of nine ROIs with highest dF/F identified by algorithm #1 **(G)** and algorithm #2 **(I)**. Orange shading, pure tone.

Most behavioral or neurophysiological experiments run in multiple trials. In these trial-based experiments, a typical trial lasts a few seconds, as does the inter-trial interval (ITI, Figure 3A). With a typical image acquisition rate of 10–30 frames/s, one trial generates a few dozen images. Adjusting optogenetic stimulation parameters based on neuronal activities in the preceding or the current trial requires identifying neurons and extracting their activities from a small set of images, and the computation must be completed within seconds. To satisfy such requirements, we developed two novel statistical algorithms based on computing temporal variance of fluorescent signals, termed *algorithm #1* (Figures 3B,C) and *algorithm #2* (Figures 3D,E), to identify active neurons within hundreds of milliseconds.

To demonstrate the online identification of active neurons, we performed *in vivo* two-photon calcium imaging in the mouse auditory cortex (ACx) and used ORCA to process the images. Each imaging trial lasted 4 s, including a 1-s “baseline” period and a 3-s “response” period. At an acquisition rate of 15 frames/s, each trial yielded 60 image frames. A 0.2-s tone was played after the baseline period (Figure 3A). As the mouse was anesthetized during imaging, shifting between frames was negligible during the 4-s imaging period, so we skipped *image registration* to further accelerate processing.

We used both algorithms of the *Mask Segmentation* module to process the images. *Algorithm #1* first computed cumulative dF/F for each pixel in the response period, and F is defined as the average intensity of each pixel during the baseline period (Figure 3B, “cumulative dF/F,” see section “Materials and Methods” for details). To separate major activities from background fluctuations, the second step was auto-thresholding of cumulative dF/F by Renyi’s Entropy (Sahoo et al., 1997) (Figure 3B, “entropy”). To segment active neurons into ROIs, the third step was additional thresholding based on user-defined ROI size and fluorescence level (Figure 3B, “labeling”). The *activity extraction* module then extracted the activity trace of each identified ROI. The whole identification process took no more than 0.3 s (Figure 3C), well within the range of inter-trial intervals for sensory and behavioral experiments. The identified neuron mask and the activity traces of 9 out of 15 identified ROIs were shown in Figures 3F,G and can be exported to downstream control systems. The ROIs were sorted by their peak dF/F in descending order.

To further speed up the processing, we designed *algorithm #2*, which first computed the standard deviation of baseline activities of all pixels from 60 images (Figure 3D, “baseline,” see section “Materials and Methods” for details), and each pixel’s fluorescence level was thresholded by its baseline. Continuous above-threshold signals were exponentially amplified (Figure 3D, “amplification”). To segment active components into ROIs, we performed thresholding based on user-defined ROI size and signal level (Figure 3D, “labeling”), and extracted the activity trace of each identified ROI using the *activity extraction* module. The whole identification process took only 0.2 s (Figure 3E), even faster than *algorithm #1*. The identified neuron mask and the activity traces of 9 out of 20 identified ROIs were shown in Figures 3H,I, sorted by their peak dF/F.

To evaluate the identification accuracy of ORCA, we used *algorithm #2* to process a publically available two-photon calcium imaging dataset from Svoboda Lab, Janelia Farm,^1^ with ground truth of labeled neurons also available. Because the dataset was not trial-based, we truncated it into 4-s long imaging windows as artificial “trials” (see section “Materials and Methods” for details). We ran ORCA on three trials and compared the results with ground truth (true labeled neurons with peak dF/F sorted from high to low, Supplementary Figure 1) and manual labeling (active ROIs identified by expert user, Supplementary Figure 2). With the default setting, ORCA achieved good performance on highly active neurons (Supplementary Figures 1C,F,I, 2C,F,I). However, ORCA missed some neurons with lower dF/F, which may be improved by fine-tuning parameters, and using a higher frame rate for imaging (see section “Materials and Methods” for details).

### Offline Segmentation of Active Neurons for Imaging Sessions

**FIGURE 4:**
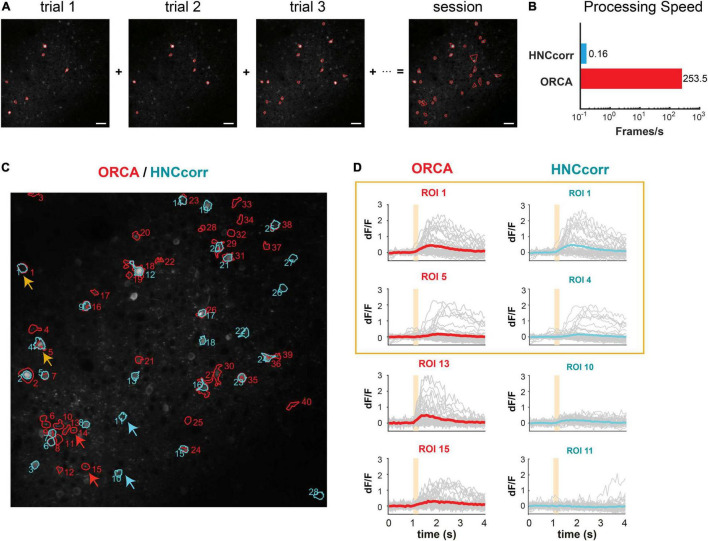
Offline active neuron identification and activity extraction for an entire session. **(A)** ROls were first identified in each trial and then integrated to generate the session mask containing all active ROIs. **(B)** Processing speed of HNCcorr and ORCA for 512 × 512 image frames. **(C)** ROI segmentation results of the same imaging session by HNCcorr (blue) vs. ORCA (red). Orange arrows: examples of ROIs identified by HNCcorr and ORCA. Red arrows: examples of ROIs identified only by ORCA but missed by HNCcorr. Blue arrows: examples of ROIs only identified by HNCcorr but missed by ORCA. **(D)** Calcium activities of arrow-pointed ROIs in panel **(C)**. ROIs 1 and 5 identified by ORCA corresponded to ROIs 1 and 4 by HNCcorr. ROIs 13 and 15 of ORCA were not identified by HNCcorr, and ROIs 10 and 11 of HNCcorr were not identified by ORCA.

For trial-based experiments, ORCA also provides a fast offline image analysis solution. An imaging session with hundreds of trials can be analyzed within minutes using the algorithms for online identification, with one extra step to integrate ROIs identified from each trial to form a unified mask for the whole session (Figure 4A). For non-trial-based experiments, we incorporated a published algorithm, HNCcorr (Speen et al., 2019), for ROI segmentation. HNCcorr can also be used for trial-based experiments. We compared the processing speed and identification accuracy of ORCA and HNCcorr in an example trial-based imaging session. In this experiment, ACx neurons were imaged while a series of different pure tones were played. From this session, ORCA and HNCcorr identified 40 and 28 ROIs, respectively, with 17 ROIs identified by both (Figures 4C,D and Supplementary Figures 1, 2). ORCA identified more ROIs than HNCcorr, including some ROIs that showed significant calcium activity (dF/F) but were missed by HNCcorr (Figures 4C,D, ORCA ROI 13 and 15; Supplementary Figure 3). ROIs that were only identified by HNCcorr but were missed by ORCA did not seem to have significant calcium activity (Figures 4C,D, HNCcorr ROI 10 and 11; Supplementary Figure 4). Thus, ORCA is more effective in identifying active ROIs. Furthermore, the processing speed of ORCA is over 1000 times faster than HNCcorr (Figure 4B, 12 s compared to 4 h, for 2,880 image frames).

### Cross-Session Alignment for Repeated Imaging

**FIGURE 5:**
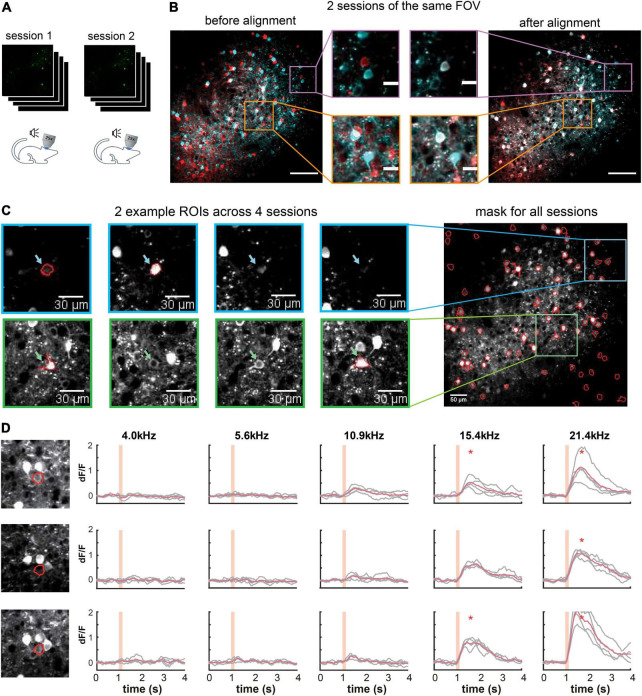
Multiple-session image alignment and data analysis. **(A)** Illustration showing multi-session imaging. **(B)** Cross-session alignment for two sessions of the same FOV. Red and cyan colors are z-project images from two imaging sessions. Zoomed-in view on the left shows displacements of neurons at both center and margins, and on the right overlapping neurons after multi-session alignment. Scale bar, 100 μm for the original image and 20 μm for the zoom-in image. **(C)** Two example ROls were identified in 2 out of 4 sessions, with similar (blue) and different (green) shapes. They were included in the all-session mask. **(D)** Calcium responses of one example ROI across three sessions. Orange shading indicates pure tone duration. *Peak activity > mean+3SD of baseline activity.

One challenge for long-term imaging is to track the same neurons across multiple imaging sessions obtained over an extended period of time (Figure 5A). Different sessions may have slightly different imaging angles due to imperfect adjustment of the microscope objective’s orientation relative to the plane of the imaging window, which hampers the identification of the same neurons across sessions. Furthermore, manual tracking is time-consuming and prone to human inconsistency. To address these issues, ORCA uses affine transformation, a linear mapping method that deals with unidirectional distortion, to correct for the angle differences between sessions (section “Materials and Methods”). An example of two overlay imaging sessions of the same field of view (FOV) is shown in Figure 5B (left panel). The same neurons from different sessions may overlap completely or partially in the overlay image if they are in the center of the FOV or not overlap if located in the periphery (Figure 5B, center panel). Our algorithm successfully aligned all the cells (Figure 5B, right panel). Masks from individual sessions were merged into one unified mask with a “capture-all” strategy: all ROIs identified in any session were kept; for ROIs identified in multiple sessions with similar (Figure 5C, blue arrow and box) or different shapes (Figure 5C, green arrow and box), the shape in the unified mask is the union of the shapes across all sessions. After indexing all ROIs in all imaging sessions, the *activity extraction* module extracts and plots the neural responses across different sessions. As an example, we plotted the individual and averaged changes in calcium signals (ÄÄ*F*/*F*) of one ROI in two imaging sessions responding to pure tones of different frequencies (Figure 5D).

### A Demo for All-Optical Closed-Loop Control of Neuronal Activities

**FIGURE 6:**
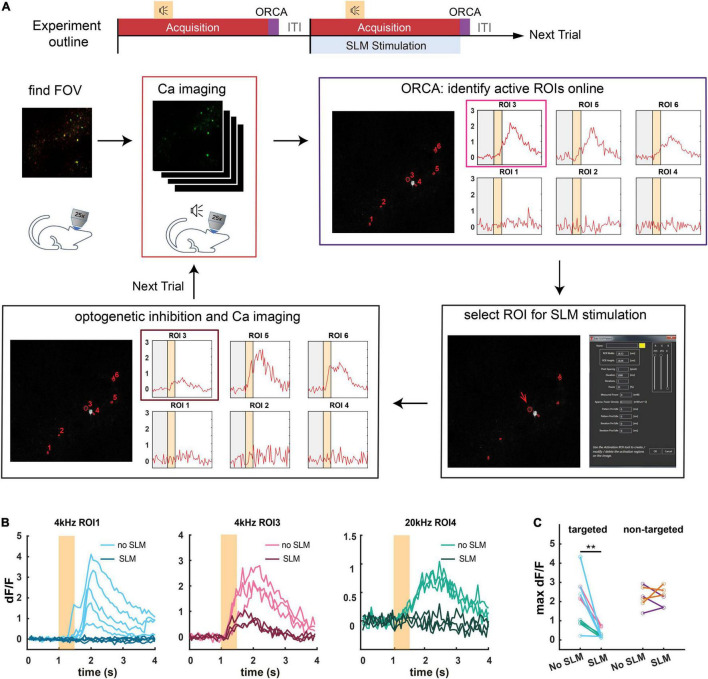
Application of ORCA in activity-based closed-loop experiments. **(A)** Timeline of the experiment. Two-photon calcium imaging is performed while different tones are played. Active ROIs are identified by ORCA online and can be selected for SLM optogenetic inhibition. **(B)** Calcium responses of three different ROIs to 4 kHz or 20 kHz pure tones in SLM optogenetic inhibition trials (SLM) and non-inhibition trials (no SLM). **(C)** Maximum dF/F for ROIs selected for optogenetic inhibition (targeted), and ROIs that were not selected (non-targeted) in optically stimulated (SLM) and unstimulated (no SLM) trials. **: *p* = 0.0012, paired *t*-test, peak activities before and after SLM stimulation.

To demonstrate ORCA’s capacity for closed-loop photostimulation, we incorporated ORCA into a Thorlabs Bergamo II two-photon microscope with a spatial light modulator (SLM) for online manipulation of tone-responsive neurons in the mouse ACx (Figure 6A). We labeled ACx neurons with genetically encoded calcium indicator and optogenetic silencer by co-injecting AAV2/9-Syn-Cre, AAV2/9-hSyn-FLEX-GCaMP6s, and AAV2/9-hSyn-DIO-hGtACR1-mCherry viruses. A subset of neurons was colabeled with GCaMP6s and GtACR1 (Supplementary Figure 5). To avoid photo-stimulation of GtACR1 while performing two-photon calcium imaging, we chose a wavelength (880 nm) that could discriminate calcium-bound GCaMP6s from calcium-free GCaMP6s and did not excite GtACR1 ((Chen et al., 2013; Govorunova et al., 2015; Mohammad et al., 2017; Mardinly et al., 2018), section “Materials and Methods”).

We delivered a variety of pure tones (0.5 s each) and imaged neuronal calcium responses starting 1 s before the tone onset for a total of 4 s per tone presentation. The ITI was 1 s, common for trial-based sensory and behavioral experiments. We used ORCA (*algorithm #1*) to identify responsive ROIs and plot calcium responses online, before the onset of the next trial (Figure 6A). By loading identified ROIs into the stimulation software (ThorImage 4.3) before each stimulation trial, we demonstrated successful targeted optogenetic inhibition using SLM (Figures 6B,C and Supplementary Figure 5) in stimulated but not control trials. The identification was proven accurate as in the stimulated trials, only the targeted ROIs were effectively inhibited, leaving the activities of the untargeted ROIs intact (Figure 6C). Thus, we showed that ORCA is well-adapted to commercial imaging and stimulation systems for activity-based closed-loop control.

## Discussion

Combining several novel algorithms, we developed ORCA, an imaging analysis toolbox that can process a small set of imaging data quickly for closed-loop neuronal stimulation, and perform cross-session image analysis for large datasets with high speed and accuracy. We validated our toolbox with calcium imaging experiments, but the software is in principle equally suitable for imaging studies using other fluorescent sensors, such as acetylcholine, dopamine, and serotonin sensors (Sun et al., 2018; Feng et al., 2019; Patriarchi et al., 2019; Wan et al., 2021).

To date, most optogenetic experiments are still open-loop, using stimulation parameters that are pre-determined rather than activity-guided. Only recently have neuronal activities been considered in guiding optogenetic manipulations, in the form of large-scale imaging data (Robinson et al., 2020) or real-time activities of pre-identified neurons (Zhang et al., 2018). Nonetheless, an imaging analysis pipeline that identifies active neurons from ongoing imaging experiments, essential for effective manipulation of highly dynamic and heterogeneous neuronal populations, is still lacking. Such a pipeline needs to complete raw image registration, active component identification, and activity extraction within a few seconds. Our GPU-based registration algorithm processes at >200 frames/s (512 × 512 pixels/frame). To our knowledge, only one method written in C++ achieves comparable speed (Mitani and Komiyama, 2018), but it requires an external OpenCV package, whereas our method is MATLAB-based and is simpler to run. For online active component identification, we designed two novel algorithms, with *algorithm #1* more sensitive to active neurons with extremely low baseline fluorescence and *algorithm #2* having a higher speed. Our method is distinguished from previous methods (Pnevmatikakis et al., 2016; Giovannucci et al., 2019; Soltanian-Zadeh et al., 2019), in that those methods require recording neuronal activity over an extended period and pre-processing before online segmentation, whereas our method can identify active neurons on the fly at >200 frames/s. This feature improves the flexibility of neuron selection, which is especially valuable for experiments involving one-trial learning such as fear conditioning and novelty detection.

Many studies have used long-term *in vivo* imaging to analyze the dynamics of the same population of neurons in multiple sessions. In such studies, neurons are typically tracked manually, which is error-prone, especially with imaging angle differences. ORCA is the first to use spatial features of the original FOV to align different sessions with possible imaging angle discrepancies. The angle difference up to 5° can be corrected. Notably, angle differences that are too large will result in data loss, especially in the margin, which cannot be fixed by post-processing. With its fast processing speed, the affine alignment function of ORCA can also be used for quality checks and calibration during image acquisition. Users can first take a small number of images, calculate the angle offset from previous sessions, and adjust the microscope accordingly to match previous recordings. As more and more microscopes feature flexible imaging angles, ORCA is a good complement and widely applicable to these systems.

Segmenting neurons from noisy *in vivo* imaging data is challenging. To achieve high accuracy, segmentation methods often have to sacrifice processing speed and rely on large quantities of imaging data. In all-optical closed-loop experiments, however, data analysis requires fast processing using only small datasets. Using novel statistical algorithms, ORCA bridges the gap and can be flexibly implemented into existing imaging systems to facilitate the application of closed-loop manipulations owing to its modular design.

## Materials and Methods

### Data Availability

The code used in this paper is available at https://github.com/YangYangLab/ORCA.

### Animals

The C57BL/6 mice were purchased from Slac Laboratory Animals (Shanghai, China). Mice were housed and bred in a 12-h light-dark cycle (7 am to 7 pm light) in the animal facility of the ShanghaiTech University. Both male and female mice were used for the experiments. All procedures were approved by the Animal Committee of ShanghaiTech University.

### Virus Injection

AAV2/9-Syn-Cre, AAV2/9-hSyn-Flpo-WPRE-pA, AAV2/9-hEFla-fDIO-GCaMP6s, AAV2/9-hSyn-FLEX-GCaMP6s, and AAV2/9-hSyn-DIO-hGtACR1-P2A-mCherry-WPRE-pA were purchased from Taitool Co., Shanghai, China. For virus injection, mice were anesthetized with isofluorane (induction, 4%; maintenance, 1–2%) and positioned onto a stereotaxic frame (Reward Co., Shanghai, China). Body temperature was maintained at 37°C using a heating pad. Viruses were injected using a glass micropipette with a tip diameter of 15–20 μm through a small skull opening (<0.5 mm^2^) with a micro-injector (Nanoject3, Drummond Scientific Co., Broomall, United States). Stereotaxic coordinates for auditory cortex (ACx): 2.46 mm posterior to the Bregma, 4.5 mm lateral from the midline, and 1.2 mm vertical from the cortical surface. For calcium imaging experiments, we mixed AAV2/9-hSyn-Flpo-WPRE-pA and AAV2/9-hEFla-fDIO-GCaMP6s with the final titer of 1.3 × 10^12^ and 2.1 × 10^12^ viral particles per ml, respectively. For closed-loop stimulation experiments, we mixed AAV2/9-Syn-Cre, AAV2/9-hSyn-FLEX-GCaMP6f-WPRE-pA, and AAV2/9-hSyn-DIO-hGtACR1-P2A-mCherry-WPRE-pA with the final titer of 6.4 × 10^9^, 6.9 × 10^12^, and 1.4 × 10^12^ viral particles per ml, respectively. We injected a 0.2-μl virus mixture into the auditory cortex for all experiments and waited 3–4 weeks before two-photon imaging experiments.

### Cranial Window Implantation

Mice were anesthetized with isoflurane (induction, 4%; maintenance, 1–2%) and positioned onto a stereotaxic frame (Reward Co.). Body temperature was maintained at 37°C using a heating pad. Lidocaine was administered subcutaneously. The muscle covering the auditory cortex was carefully removed with a scalpel. A 2 × 2 mm^2^ piece of the skull over ACx was removed, exposing the dura. The cranial window was sealed with a custom-made double-layered cover glass. UV-cure glue and dental acrylic were used to cement the cover glass onto the skull. A custom-made stainless steel head plate with a screw hole was embedded into the dental acrylic for head-fixed two-photon imaging.

### Two-Photon Calcium Imaging and Optogenetic Stimulation Using the Spatial Light Modulator

Mice were injected with pentobarbital sodium (20 mg/kg) and head-fixed using the implanted head plate. Image series were taken at 15 Hz with a two-photon microscope (Bergamo II, Thorlabs, Newton, NJ, United States) equipped with a 25X/NA 1.05 objective (Olympus, Kyoto, Japan) and a Ti:sapphire laser (DeepSee, Spectra-Physics, Santa Clara, CA, United States) tuned to 880 nm. We chose this wavelength rather than the optimal wavelength for imaging GCaMP6s (930 nm) to avoid exciting GtACR1 during imaging. Mice were imaged when a series of pure tones (4, 12, and 20 kHz) were played. The sound was controlled using a Bpod state machine (Sanworks Co., Rochester, NY, United States) and open-source Bpod software (Sanworks Co.).

A fixed-wavelength laser (1,040 nm, Spectra-Physics, United States) and spatial light modulator (Thorlabs) were used for two-photon targeted optogenetic silencing. A cycle of 40 was used for inhibiting selected ROIs (0.1 s per cycle). The SLM was controlled by ThorImage (Thorlabs) and the control module of ORCA.

### Implementation of Online Real-Time Activity and Offline Cross-Session Analysis to Microscope Software

We used ThorImage 4.0 (Thorlabs) for two-photon imaging and SLM stimulation. ORCA was implemented by adding an output interface to ThorImage 4.0 for acquiring two-photon imaging data, and an input interface to the stimulation module of ThorImage 4.0 for sending identified ROIs after image processing, with help from Thorlabs software engineers.

### Hardware for Imaging Data Analysis

We used a Dell workstation to perform all computations. The workstation is equipped with two Intel Xeon Gold 5122 CPU, 64 GB DDR4 RAM, and an Nvidia Quadro P6000 GPU, running Linux Debian 10. ORCA uses GPU to accelerate most processing steps, while CPU versions are also provided in the package. We recommend the GPU version for best performance.

### Preparation and Evaluation of Image Registration

To compare the speed of different algorithms, we performed registration in their default environments: single-step DFT and ORCA were tested in MATLAB R2020a (MathWorks, Natick, MA, United States), while TurboReg and moco in Fiji (ImageJ 1.48v, Java 1.6.0_24, 64bit). File read/write time was excluded for a better evaluation of the computational efficiency of each algorithm. Timing of TurboReg, single-step DFT, and ORCA started after files were loaded into the memory and stopped as soon as algorithms produced registered results. The timing of moco was evaluated using a screen recording software (Kazam Screencaster) and inspected manually.

To quantitatively compare different algorithms, we generated a simulated movie as follows: An example movie consisting of 1,200 frames was repeatedly registered using moco, TurboReg (accurate mode), and ORCA for 10 times each. This movie was then carefully examined by tracking major features in the images and measuring their movements frame-by-frame, to ensure it reached maximum possible stability. Then, periodical shifts caused by heartbeats were simulated by adding directional movements using the following equation:


$$s_{x,i}=\left\{ \begin{array}{c} 0,\sin\left(\frac{2\,\pi }{F}\,i\right)<0\\ M_{x}*\sin\left(\frac{2\,\pi }{F}\,i\right),\sin\left(\frac{2\,\pi }{F}\,i\right)\geq 0 \end{array}\right.$$

where *F* indicated frames-per-second during acquisition, *M_x_* the maximum shifts in x-direction, and *i* the current frame count. This kept all positive values in *s_x_* and set all negative offsets to zero. An additional random distortion was also introduced using pseudo-random numbers as offsets. Thus, shifts in x-direction, *s_x_*, became


$$s_{x,i}=\left\{ \begin{array}{c} \text{rand}\,\left(-M_{r},M_{r}\right),\sin\left(\frac{2\,\pi }{F}\,i\right)<0\\ M_{x}*\sin\left(\frac{2\,\pi }{F}\,i\right)+\text{rand}\,\left(-M_{r},M_{r}\right),\sin\left(\frac{2\,\pi }{F}\,i\right)\geq 0 \end{array}\right.$$

where rand(−*M_r_*, *M_r_*) generated a pseudo-random sequence ranging from [−*M*_*r*_, *M*_*r*_].

True shift values were generated in both x and y directions in the same manner with varying parameters, and a simulated movie was tested among all four registration methods.

Registration accuracy was quantified as


$$\text{diff}\,\left(t,e\right)=\sqrt{\sum_{i=1}^{n}{\left(t_{i}-e_{i}\right)}^{2}}$$

where *t* was the true values and *e* was the estimated shifts generated by each algorithm. The registration accuracy was calculated separately for the x and y directions.

### Image Registration Algorithms

Inspired by the moco algorithm (Dubbs et al., 2016), we combined multiple computational ideas to produce a faster implementation. Let *a* be an image frame of *h* rows (height) and *w* columns (width), and *a*_*i,j*_ denote the pixel of the *i*th row, *j*th column. Let *t* be the template image (usually the first frame in an image series). Images *t* and *a* are standardized by mean-centering before registration:


$$a:=\frac{a-\bar{a}}{\text{std}\,\left(a\right)}$$

$$t:=\frac{t-\bar{t}}{\text{std}\,\left(t\right)}$$

Let *Ms* be the user-defined maximum shift value. We set its default value to 1/5 of the frame size. Users are advised to start with the default setting and adjust the parameter from there, as an appropriate choice of *Ms* is important for the best performance. An *Ms* too large (e.g., for a 512 × 512 pixel image, *Ms* = 170) may result in faulty matches, while an *Ms* too small (e.g., for a 512*512 image, *Ms* = 1) will cause the registration to fail, because the x–y shift between frames is likely to exceed 1 pixel.

We search for the optimal *x*, *y* offsets within the maximum shifts to minimize the difference between *a* and *Tm* in their overlapping region, defined as


$$D\,\left(x,y\right):=\frac{1}{\left(h-\left|x\right|\right)\,\left(w-\left|y\right|\right)}\,\sum_{i,j}{\left(t_{i,j}-a_{i+x,j+y}\right)}^{2}$$

Substituting the denominator (*h*−|*x*|)(*w*−|*y*|) with *A*_*x,y*_, the area of overlap between the template and the target frame, we can rewrite the equation as:


$$A_{x,y}\cdot D_{x,y}=\sum_{i+x,j+y}t_{i+x,j+y}^{2}+\sum_{i,j}a_{i,j}^{2}-2\,\sum_{i,j}a_{i,j}\cdot t_{i+x,j+y}$$

in which *D*_*x,y*_ is the sum of all pixels squared in the overlapping area of the template, and *i*, *j* are corresponding indices. Given that *x*, *y* ∈ [−*ms*, *ms*]∩ℤ, there are finite possibilities of these values, and they can be calculated for any given *x*, *y* in the range beforehand.

To efficiently calculate these values, we register template *t* onto current frame *a*, so that ∑*t*^2^ only need to be calculated once throughout the whole registration process. ∑*a*^2^ and ∑*a*⋅*t*, are both related to the current frame *a*. Notice that, once we determined the maximum shift *ms*, only a central fraction of the original images are then registered to the template. To further accelerate the computation, we crop out the marginal areas of the original image, using only the central part to register against the full template. The cropped image, *a*′, should be


$$a^{\prime }:=a_{i^{\prime },j^{\prime }}$$

Where *i*′ ∈ [*Ms*,*h*−*Ms*], and *j*′ ∈ [*Ms*,*w*−*Ms*]. Thus we rewrite the equation into:


A′⋅Dx,y=∑i+x,j+yti+x,j+y2+∑i,ja′i,j2-2⁢∑i,ja′i,j⋅ti+x,j+y

In this equation, *a*′ are the same cropped image for any valid *x*, *y* ∈ [−*ms*, *ms*]∩ℤ and the overlapping area *A*′ becomes static. The only dynamic part in the equation thus becomes ∑*a*⋅*t*. ∑*a*⋅*t* is the convolution of *a* and $\tilde{\text{t}}$ evaluated at (0,0). Letting $\tilde{\text{t}}$ be the rotation of *t* by 180 degrees, we have


$$\sum_{i,j}a_{i,j}\cdot t_{i+x,j+y}=\mathrm{conv2}\,\left(a,\tilde{\text{t}}\right)$$

Convolution can be efficiently calculated using two-dimensional fast Fourier transform (2D-FFT):


$$\mathrm{conv2}\,\left(a,\tilde{\text{t}}\right)=\mathrm{ifft2}\,\left(\mathrm{fft2}\,\left(a\right)\cdot \mathrm{fft2}\,\left(\tilde{\text{t}}\right)\right)$$

and thus we developed a highly optimized conv2 function (conv2_fftvalid.m in the source code) for our registration, which is much faster than MATLAB’s default implementation using basic addition and multiplication.

∑*a*^2^ can also be calculated as


$$\sum_{i,j}a_{i,j}^{2}=\mathrm{conv2}\,\left(a^{2},\text{ones}\,\left(h-2\,M\,s,w-2\,M\,s\right)\right)$$

where ones(*h*−2*Ms*, *w*−2*Ms*)) is a matrix of size (*h*−2*Ms*, *w*−2*Ms*) with all elements being 1, in the MATLAB notation.

The whole registration process can be further accelerated by downsampling and doing a local search after upsampling. Running on GPU, our code is much faster than most other registration algorithms.

### Online Trial-By-trial Identification of Active Neurons

#### Algorithm #1

We combined several statistical tools for online segmentation of active ROIs on a trial-by-trial basis, each trial consisting of *N* frames of size (*H*, *W*) that constitute an image stack.

The dF/F (here denoted by ${\tilde{s}}_{i,j}$for pixel (*i*, *j*)) is computed by subtracting the baseline intensity (${\bar{s}}_{i,j}$) of each pixel in the image stack and divided by ${\bar{s}}_{i,j}$:


$${\bar{s}}_{i,j}:=\frac{1}{N}\,\sum_{t}s_{i,j}\,\left(t_{k}\right),t_{k}\in t_{b\,l}$$

$${\tilde{s}}_{i,j}=\frac{s_{i,j}-{\bar{s}}_{i,j}}{{\bar{s}}_{i,j}}$$

where *s*_*i*, *j*_(*t*) represents a pixel (*i*, *j*) of the *t*th image in the stack and *t*_*bl*_ is the baseline period (defined by the user). Then, the dF/F of each pixel is summed up to form a two-dimensional time-average image. Values below zero are substituted by zeros.


$${\tilde{v}}_{i,j}:=\text{max}\,\left(0,\sum_{t}{\tilde{s}}_{i,j}\right)$$

Here, ${\tilde{v}}_{i,j}$ represents the cumulative dF/F for pixel (*i*, *j*) in the 2-D image. To increase the signal-to-noise ratio, we use the standard deviation of ${\tilde{s}}_{i,j}$ as a scaling factor, and multiply by ${\tilde{v}}_{i,j}$:


$$v_{i,j}:={\tilde{s}}_{i,j}\cdot {\tilde{v}}_{i,j}$$

The product matrix *v*_*i*,*j*_undergoes a filter that converts it to a binary image using Renyi Entropy-based auto thresholding (Sahoo et al., 1997) by calling the MIJ plugin in Fiji (Daniel Sage et al., 2012). The binary image is smoothed using a Gaussian filter to combine neighboring pixels. Two additional user-defined thresholds can be applied to determine whether to keep the identified ROIs (area threshold, default set at 16 pixels; intensity threshold, default set to 10). Pixels containing 1 (“positive pixels”) indicate a masked region.

#### Algorithm #2

The stack is first binarized to zeros and ones with a brightness detection threshold of 3-fold standard deviation over the average of baseline pixels:


$$d_{i,j}:=\sqrt{\frac{\sum {\left(s_{i,j}-{\bar{s}}_{i,j}\right)}^{2}}{N}}$$

$$\chi_{i,j}:=\left\{ \begin{array}{c} 1,\text{if}\,s_{i,j}>{\bar{s}}_{i,j}+3\,d_{i,j}\\ 0,\text{if}\,s_{i,j}\leq {\bar{s}}_{i,j}+3\,d_{i,j} \end{array}\right.$$

where *d*_*i,j*_ represents the detection threshold, and χ is a step function representing the binarized stack, here we call binary sensitivity index. Fluorescent calcium signals generated by neural activities, rather than random noise, will be continuously above the threshold in the time domain, generating continuous ones in χ_*i*,*j*_. On the other hand, random noises may go above and below the detection threshold in a stochastic manner, generating alternating ones and zeros. We, therefore, apply an exponential-like filter on this binary sensitivity index to amplify continuous ones:


$$L_{t}=\left\{ \begin{array}{c} \alpha \,\chi_{t}\,\left(L_{t-1}+\beta \right)\,\chi_{t},\text{if}\,t\geq 2\\ \chi_{t},\text{if}\,t=1 \end{array}\right.$$

Here, α, β are user-defined amplifying coefficients, and by default β = α^−1^. Continuous ones in the stack are amplified exponentially, while noise signals remained low in the temporal trace. This filter is then applied to the binary stack χ_*t*_ and summed up the results in the temporal trace to get a sensitivity index image *L*. This image *L* summarized the degree to which each pixel may contain fluorescent activity beyond noise level. We smoothed *L* with a gaussian (σ=1) convolution kernel of the cell-sized window and applied a threshold related to fluorescent indicator dynamics:


$$T=\alpha^{F}+k$$

where *F* is the dynamic time of the fluorescent indicator used, which tells the algorithm how many continuous frames above the detection threshold should be considered a valid activity, and can be adjusted accordingly. Because the algorithm favors larger numbers of continuous above-threshold frames, it is much more suitable for imaging data acquired with high frame rates. *k* is a parameter that arbitrarily adjusts threshold T and is set to 0 by default. Connected areas in the final mask (using MATLAB bwconncomp function) after thresholding are used as estimates of areas with neural activity.

### Benchmarking Using a Public Dataset

We downloaded a publically available two-photon imaging dataset from NeuroFinder^2^ (data from Svoboda Lab, Janelia Farm), and truncated 4-s imaging windows as artificial “trials” for online neuronal segmentation. We chose three “trials” for benchmarking, the criteria being: (1) the 1-s “baseline” is relatively flat and (2) there are some calcium activities in the 3-s “response” period. Since the ROIs of all labeled neurons were also available on the website, we extracted the dF/F of all labeled neurons and sorted them by peak dF/F (top 20 shown in Supplementary Figures 1C,F,I for three trials). For manual annotation, the first author of this paper hand-drew ROIs with identifiable fluorescence blind to the ground truth and ORCA identification results. ROIs were sorted by peak dF/F and the top 20 were shown in Supplementary Figures 2C,F,I. False-negative rate can also be reduced by fine-tuning the parameters, such as reducing *F*, or increasing the imaging frame rate (such as 15 fps or higher).

### Offline Manual Segmentation of Components in a Movie

Since automated identification may falsely identify unwanted components or neglect true active neurons, we provide a user-friendly interface for manual labeling of active neurons in a given movie, incorporated in the mask_segmentation module. Our manual labeling program allows users to play the movie for full length or just one trial, and add or erase components.

### Cross Session Alignment

For multi-session imaging data of one FOV, we use time-averaged images to represent the imaging sessions and define the time-average image of the first imaging session as the reference image. In three-dimensional space, we define the xy plane as the plane of the reference image, and z-axis perpendicular to xy. We then define the target image to be the time-average of a different imaging session. For sessions without rotation on the z-axis, displacements in x- and y-axis were corrected simply by moving target images in x and y directions for optimum alignment. For sessions with rotation, because the image plane preserves the collinearity of the FOV, displacements can be corrected using affine transformation. We first computed similarity between the reference image and the target image, then applied different transformations to the target image and search for maximum similarity between them. We used MATLAB function imregtform() with “rigid” or “affine” parameters and applied the transformation matrix by the function. We incorporated manual inspection and refinement by the users in this module. After user confirmation of the alignment results, masks with segmented ROIs from different sessions were combined to generate a superset of ROIs. These ROIs were then transformed to the original session based on respective transformation matrices. ROIs in the margins may be cropped out in the cross-session alignment.

### Activity Extraction and Additional Thresholding

As ORCA extracts neuronal activity trial-by-trial, the user needs to supply information about the trial structure, in particular, timing parameters. The baseline activity *F_0_* of each ROI is the average of all pixels over a user-defined time window before stimulus onset:


$$F_{0}:=\frac{\sum_{i,t}a_{i}\,\left(t\right)}{i\cdot t}$$

where *a_i_* is a pixel in the corresponding ROI and *t* ∈ [*BaselineFrames*]. Then, the normalized activity of all ROIs is calculated by subtracting and dividing baseline activities:


$$F:=\frac{\sum_{i}a_{i}}{n},F_{a\,c\,t\,i\,v\,i\,t\,y}\,\left(t\right):=\frac{F\,\left(t\right)-F_{0}}{F_{0}}$$

By default, an ROI is considered active if its peak activity rises above five standard deviations than the mean baseline activity $\bar{F_{0}}$ (Dombeck et al., 2007; O’Connor et al., 2010). Active ROIs are then sorted based on their peak *F*_*activity*_ activities, and plotted or exported to a closed-loop controlling system.

Users can use other criteria for defining active ROIs, by (1) manually choosing a dF/F threshold, and ROIs with peak activity *F*_*activity*_ lower than this threshold will be discarded; or (2) manually setting a user-defined minimum ROI size to exclude smaller ROIs.

## Data Availability Statement

The raw data supporting the conclusions of this article will be made available by the authors, without undue reservation.

## Ethics Statement

The animal study was reviewed and approved by Animal Committee of ShanghaiTech University.

## Author Contributions

WS and YY conceived the project. WS, XZ, and XH performed the experiments. WS developed the algorithms. WS and YY wrote the manuscript with contributions from all authors.

## Conflict of Interest

The authors declare that the research was conducted in the absence of any commercial or financial relationships that could be construed as a potential conflict of interest.

## Publisher’s Note

All claims expressed in this article are solely those of the authors and do not necessarily represent those of their affiliated organizations, or those of the publisher, the editors and the reviewers. Any product that may be evaluated in this article, or claim that may be made by its manufacturer, is not guaranteed or endorsed by the publisher.

## Footnotes

1
http://neurofinder.codeneuro.org/

2
http://neurofinder.codeneuro.org/

## Supplementary Material

Supplementary Figure 1 Benchmarking with the public dataset: comparing with ground truth. **(A,D,G)** ROIs identified by ORCA (red) and true-labeled neurons (blue). ROIs and neurons were sorted by peak dF/F (high to low). For demonstration purposes, 20 identified ROIs and 20 labeled neurons with the highest dF/F were shown. **(B,E,H)** Calcium responses of the 20 most active ROIs identified by ORCA. Black bar separates “baseline” and “response” periods of truncated artificial “trials.” **(C,F,I)** Calcium responses of the 20 most active-labeled neurons. Red shadings indicate neurons identified by ORCA.

Supplementary Figure 2 Benchmarking with the public dataset: comparing with manual annotation. **(A,D,G)** ROIs identified by ORCA (red) and manually by expert user (blue). ROIs were sorted by peak dF/F (high to low). For demonstration purposes, 20 identified ROIs with the highest dF/F were shown. **(B,E,H)** Calcium responses of the 20 most active ROIs identified by ORCA. Black bar separates “baseline” and “response” periods of truncated artificial “trials.” **(C,F,I)** Calcium responses of the 20 most active ROIs identified manually. Red shadings indicate ROIs also identified by ORCA.

Supplementary Figure 3 Calcium responses of each ROI identified by ORCA shown in Figure 4C.

Supplementary Figure 4 Calcium responses of each ROI identified by HNCcorr shown in Figure 4C.

Supplementary Figure 5 Expression of hGtACR1-mCherry (red) and GCaMP6s (green) in the FOV shown in Figure 6. Scale bar, 50 μm.
