# Supplementary material for: Real-Time Image Processing Toolbox for All-Optical Closed-Loop Control of Neuronal Activities
Source: Front Cell Neurosci. 2022 Jul 5;16:917713. doi: 10.3389/fncel.2022.917713 (PMC9294372; doi:10.3389/fncel.2022.917713)

Supplementary Figure legends

Supplementary Figure 1

Benchmarking with public dataset: comparing with ground truth.

(a,d,g) ROIs identified by ORCA (red) and true labeled neurons (blue). ROIs and neurons were sorted by peak dF/F (high to low). For demonstration purpose, 20 identified ROIs and 20 labeled neurons with the highest dF/F were shown.

(b,e,h) Calcium responses of the 20 most active ROIs identified by ORCA. Black bar separates “baseline” and “response” periods of truncated artificial “trials”.

(c,f,i) Calcium responses of the 20 most active labeled neurons. Red shadings indicate neurons identified by ORCA.

Supplementary Figure 2

Benchmarking with public dataset: comparing with manual annotation.

(a,d,g) ROIs identified by ORCA (red) and manually by expert user (blue). ROIs were sorted by peak dF/F (high to low). For demonstration purpose, 20 identified ROIs with the highest dF/F were shown.

(b,e,h) Calcium responses of the 20 most active ROIs identified by ORCA. Black bar separates “baseline” and “response” periods of truncated artificial “trials”.

(c,f,i) Calcium responses of the 20 most active ROIs identified manually. Red shadings indicate ROIs also identified by ORCA.

Supplementary Figure 3

Calcium responses of each ROI identified by ORCA shown in Figure 4c.

Supplementary Figure 4

Calcium responses of each ROI identified by HNCcorr shown in Figure 4c.

Supplementary Figure 5

Expression of hGtACR1-mCherry (red), and GCaMP6s (green) in the FOV shown in Figure 6. Scale bar, 50 μm.

Supp. Figure 1

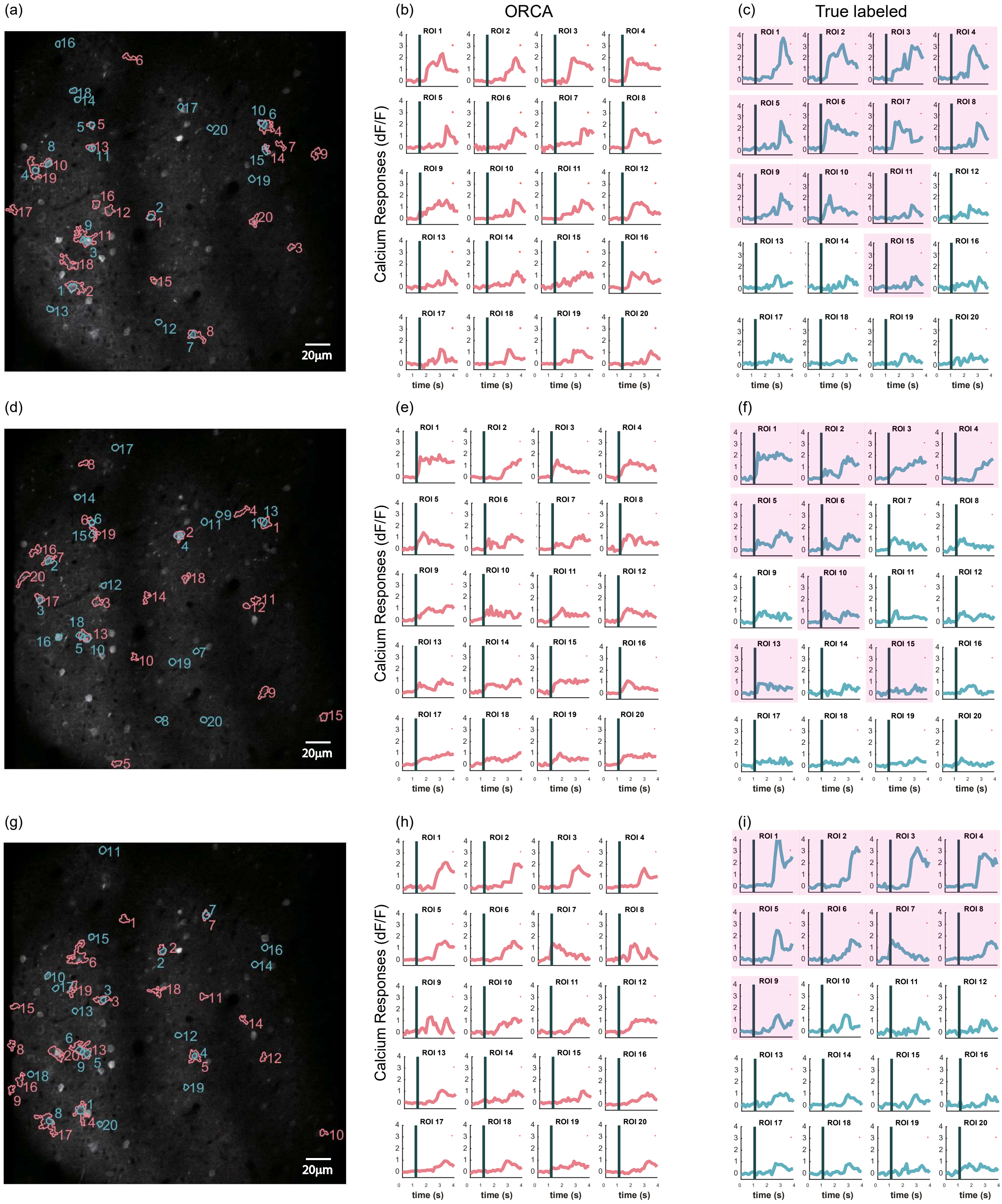

Supp. Figure 2

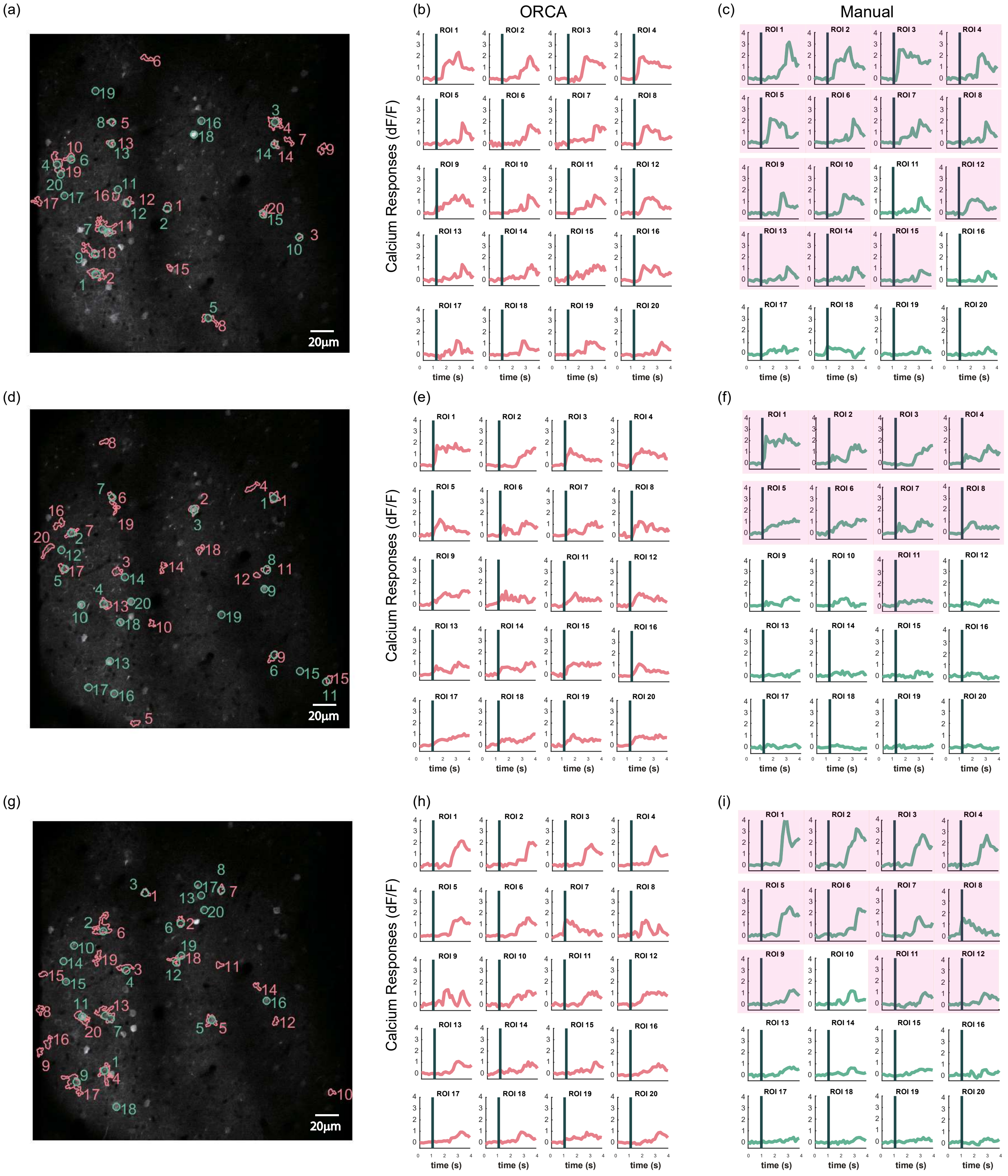

Supp. Figure 3

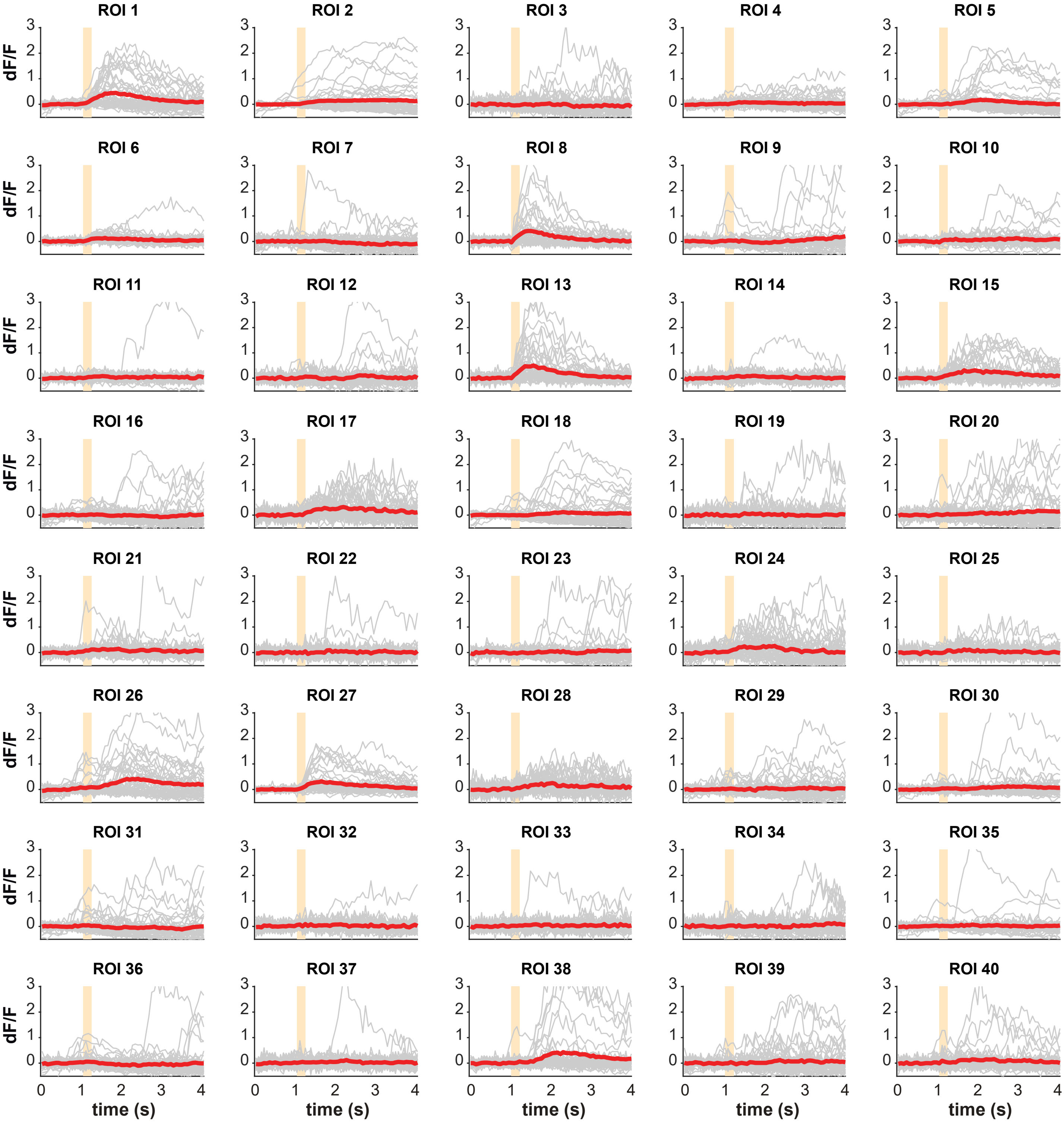

Supp. Figure 4

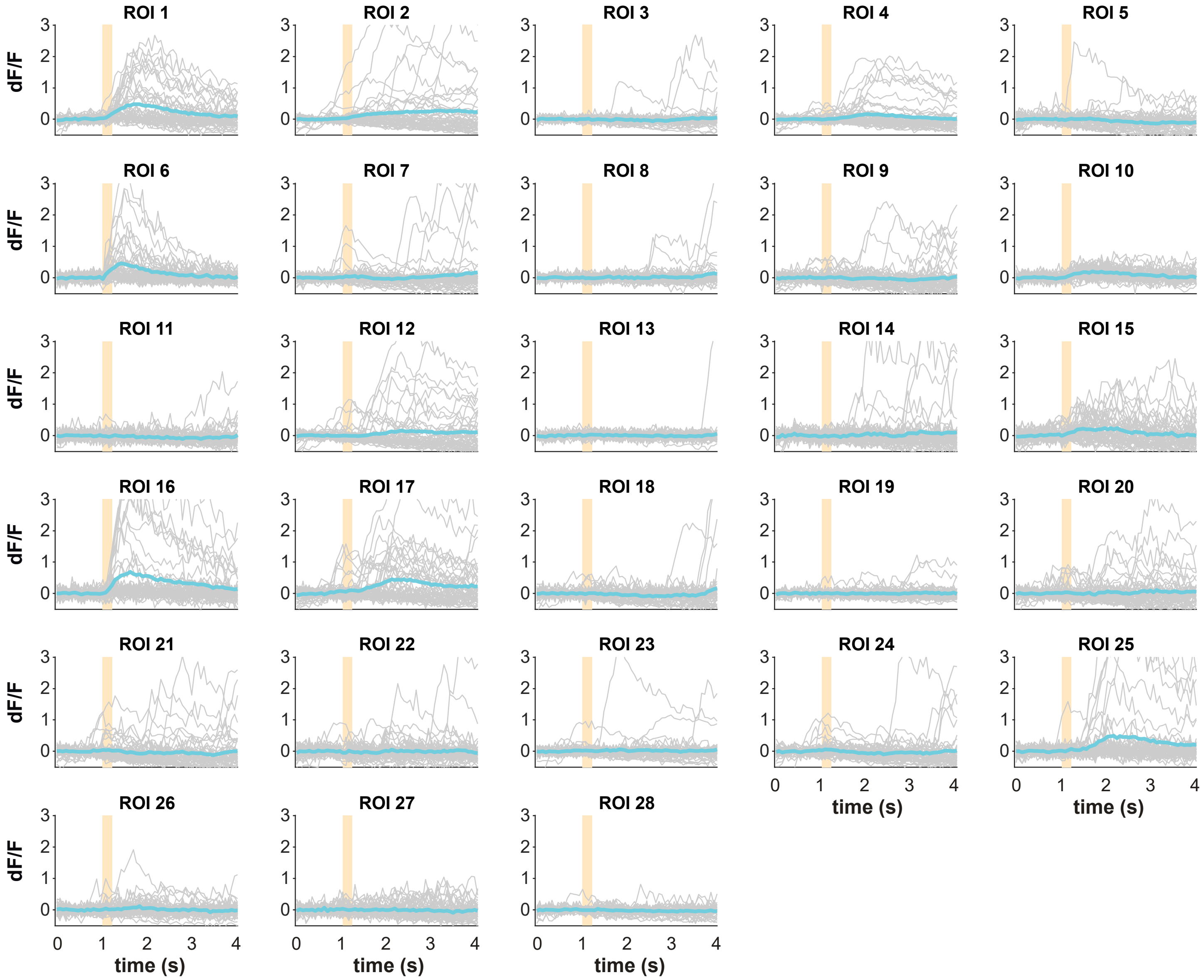

Supp. Figure 5

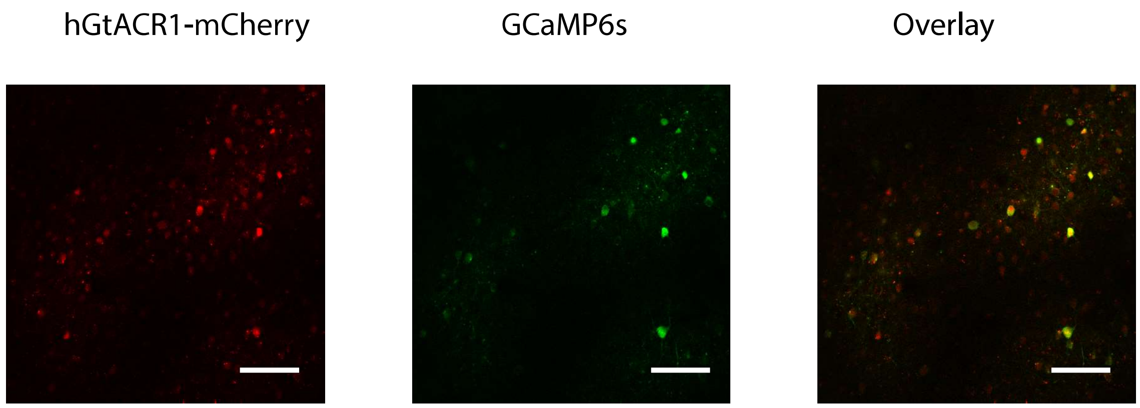

Supplement: Supplementary Figure 1 — Benchmarking with the public dataset: comparing with ground truth. (A,D,G) ROIs identified by ORCA (red) and true-labeled neurons (blue). ROIs and neurons were sorted by peak dF/F (high to low). For demonstration purposes, 20 identified ROIs and 20 labeled neurons with the highest dF/F were shown. (B,E,H) Calcium responses of the 20 most active ROIs identified by ORCA. Black bar separates “baseline” and “response” periods of truncated artificial “trials.” (C,F,I) Calcium responses of the 20 most active-labeled neurons. Red shadings indicate neurons identified by ORCA. [file Data_Sheet_1.pdf]
